# Fractionated Repetitive Extracorporeal Shock Wave Therapy: A New Standard in Shock Wave Therapy?

**DOI:** 10.1155/2015/454981

**Published:** 2015-07-27

**Authors:** Tobias Kisch, Heiko Sorg, Vinzent Forstmeier, Peter Mailaender, Robert Kraemer

**Affiliations:** ^1^Department of Plastic Surgery, Hand Surgery, Burn Unit, University Hospital Schleswig-Holstein, Campus Lübeck, University of Lübeck, 23538 Lübeck, Germany; ^2^Department for Plastic, Reconstructive and Aesthetic Surgery, Hand Surgery, Alfried Krupp Hospital, 45276 Essen, Germany; ^3^Department of Visceral and Thoracic Surgery, German Armed Forces Hospital Ulm, 89081 Ulm, Germany

## Abstract

*Background*. ESWT has proven clinical benefit in dermatology and plastic surgery. It promotes wound healing and improves tissue regeneration, connective tissue disorders, and inflammatory skin diseases. However, a single treatment session or long intervals between sessions may reduce the therapeutic effect. The present study investigated the effects of fractionated repetitive treatment in skin microcirculation. *Methods*. 32 rats were randomly assigned to two groups and received either fractionated repetitive high-energy ESWT every ten minutes or placebo shock wave treatment, applied to the dorsal lower leg. Microcirculatory effects were continuously assessed by combined laser Doppler imaging and photospectrometry. *Results*. In experimental group, cutaneous tissue oxygen saturation was increased 1 minute after the first application and until the end of the measuring period at 80 minutes after the second treatment (*P* < 0.05). The third ESWT application boosted the effect to its highest extent. Cutaneous capillary blood flow showed a significant increase after the second application which was sustained for 20 minutes after the third application (*P* < 0.05). Placebo group showed no statistically significant differences. *Conclusions*. Fractionated repetitive extracorporeal shock wave therapy (frESWT) boosts and prolongs the effects on cutaneous hemodynamics. The results indicate that frESWT may provide greater benefits in the treatment of distinct soft tissue disorders compared with single-session ESWT.

## 1. Introduction

### 1.1. Extracorporeal Shock Wave Therapy

In the 1980s, the advent of shock wave lithotripsy in urology led to widespread awareness of extracorporeal shock waves [1]. This method was defined as a sequence of sonic pulses characterized by high peak pressure with fast pressure rise and short life cycle. Later, extracorporeal shock wave treatment (ESWT) showed excellent results in orthopedics, especially when used to treat tendinopathies and chronic epicondylitis [2]. ESWT has an impact on erectile dysfunction due to a significant improvement of hemodynamics [3]. Moreover, ischemic tissues have been shown to benefit from ESWT, for example, ischemic flaps survived [4–6] and myocardial ischemia, limb ischemia, and osteonecrosis were reduced [7–9]. Fracture healing [10] and acute and chronic wound healing were enhanced [11–13] and even ischemia reperfusion injury was reduced [14]. The described effects were explained by an increase of blood flow and angiogenesis in the treated tissue.

### 1.2. Influencing Dermal Connective Tissue

After skin smoothening effects of ESWT had been demonstrated, first studies were conducted to evaluate its use for conditions associated with hypertrophic scar formation and connective tissue disorders. In a recent study, it was shown that burn scars appearance was more soft and movable after ESWT using 0.037 mJ/mm^2^ with 100 impulses twice a week for six weeks. As a result, range of motion, especially in neck areas, was improved [15]. A randomized, controlled trial including 53 patients found that the use of focused ESWT in combination with gluteal strength training improved moderate to severe cellulite after 12 weeks of treatment. Patients in the experimental group received 0.35 mJ/mm^2^ with 2,000 impulses every 1-2 weeks in six sessions [16]. Cellulite skin bulging results from an imbalance between lipogenesis and lipolysis in combination with the connective tissue aging. Improvements in the study were explained by direct effects on the associated lymphedema [17] in combination with enhanced skin elasticity, dermal revitalization, and smoothness by disruption of fat components and fibrous septae [18].

### 1.3. Inflammatory Skin Diseases

Several studies indicate that ESWT has an effect on tissue inflammation by suppressing proinflammatory factors [19–21]. In a clinical trial, panniculitis areas resulting from interferon *β* or glatiramer acetate application in multiple sclerosis patients were reduced by the delivery of 0.25 mJ/mm^2^ with 2,000 impulses. The authors found a decrease of indurations and a softer and more elastic skin at the treated injection sites. ESWT application was performed once a week for a cycle of 5 sessions [22].

These data indicate that repeated application of ESWT may have beneficial effects on various skin conditions. However, long intervals between applications may decrease the possible benefit. Moreover, reasons for additive effects of repetitive ESWT have not yet been evaluated.

Therefore, we applied fractionated repetitive ESWT (frESWT) in an established rat model to evaluate additive effects on microcirculation of the skin.

## 2. Methods

### 2.1. Animal Model and Experimental Protocol

Thirty-two Sprague Dawley rats (Charles River Laboratories, Sulzfeld, Germany), weighing 250 to 350 g, were used in this study. Animals were housed in 12 hours per day/night cycle, 2/cages at 21°C and fed ad libitum. The experimental procedures were conducted in accordance with the German legislation on protection of animals and the National Institutes of Health Guide for the Care and Use of Laboratory Animals (Institute of Laboratory Animal Resources, National Research Council).

Rats were randomly assigned to the active treatment and the placebo groups (*n* = 16 each). Experimental group A received fractionated repetitive high-energy ESWT every ten minutes (shock waves at 3 × 0.3 mJ/mm^2^ and 4 impulses/s with a total of 1000 impulses totaling 30 J). Placebo group B received placebo ESWT without energy application (0 impulses totaling 0 J). ESWT was applied to the dorsal lower leg of the hind limb of each animal with a short range applicator (focus 0–30 mm) using a Storz Medical Duolith SD-1 “T-Top.” Application was standard for both groups and performed by the same physician using contact gel without relevant pressure to the tissue. During the experiments, the rats were under sufficient pentobarbital sodium anesthesia (55 mg/kg bw ip; Narcoren, Merial, Hallbergmoos, Germany), monitored by stable heart rate and breathing frequency, in order to minimize microcirculatory reactions in response to pain. Body temperature was maintained at 36-37°C using a heating pad.

### 2.2. Microcirculatory Analysis

The cutaneous microcirculation of each rat was allowed to stabilize for 10 minutes after anesthesia delivery prior to investigating the cutaneous microcirculation. Microcirculation was assessed at the lower leg of the hind limb before application and 1, 2, 5, and 10 minutes after the first and the second application and 1, 2, 5, 10, 15, 20, 30, and 60 minutes after the third application of ESWT, using a noninvasive combined laser Doppler and photospectrometry system (Oxygen-to-see, O2C, LEA Medizintechnik, Giessen, Germany).

In order to minimize measurement artifacts due to vibration, the probe was fixed by a special apparatus. One minute before application of focused ESWT, baseline measurements were carried out on the dorsal lower leg of the hind limb of each animal.

The O2C system combines the hemoglobin measurement and the principle of blood flow measurement. As described before [23], the optical method allows measuring both blood flow velocity using laser Doppler imaging technology and hemoglobin oxygenation and hemoglobin concentration in the tissue using photospectrometric techniques. In short, an optical fiber probe, incorporating both the laser Doppler and the broadband light spectrometry technique, records the local oxygen supply parameters, the oxygen saturation of hemoglobin, the relative postcapillary venous filling pressures, and the blood flow velocity. It uses assessed data in a 200 *μ*m depth with respect to cutaneous tissue oxygen saturation (%), cutaneous venous filling pressure (AU), and cutaneous capillary blood flow velocity (arbitrary units; AU).

### 2.3. Statistical Analysis

Data was analyzed using SigmaPlot statistical software version 12.3 (Systat, San José, USA). Normal distribution was tested using the Shapiro-Wilks test. In case of parametric data, statistical analysis was carried out using ANOVA followed by Tukey's test. In case of nonparametric data, ANOVA on ranks was used followed by Dunn's test. Data were expressed as mean ± SD. *P* values less than 0.05 were regarded as statistically significant.

## 3. Results

The animals tolerated the treatment very well and did not show any deviant behavior or pain after anesthesia. Moreover, no petechiae, hematoma, seroma, or bleeding was observed.

### 3.1. Cutaneous Tissue Oxygen Saturation (Figure 1)

Baseline cutaneous tissue oxygen saturation was similar in both groups (experimental group: 48.3 ± 12.8% and placebo group: 49.8 ± 12.4%, *P* > 0.05). Tissue oxygen saturation remained statistically unchanged from baseline until 80 minutes in the placebo group. In the experimental group, tissue oxygen saturation significantly increased by 65.6% (percentage change) one minute after the first application of ESWT (80.0 ± 11.1%; *P* < 0.05) and remained on an elevated level until the second ESWT application. The third application of ESWT boosted tissue oxygen saturation to the highest extent of 90.5 ± 7.4% (*P* < 0.05) and remained significantly increased until the end of measuring at 80 minutes (82.9 ± 14.5%; *P* < 0.05).

### 3.2. Postcapillary Venous Filling Pressure (Figure 2)

Baseline cutaneous postcapillary venous filling pressure in the experimental group and placebo group is similar (experimental group: 25.9 ± 9.8 arbitrary units [AU]; placebo group: 22.1 ± 3.6 AU, *P* > 0.05). Postcapillary venous filling pressure in the experimental group increased 1 minute after each application of ESWT (first application: 36.6 ± 9.4; second application: 37.4 ± 11.3; third application: 36.5 ± 11.5), statistically insignificant, respectively, decreasing to baseline 20 minutes after application (27.7 ± 8.8). The placebo group showed no significant changes from the baseline either.

### 3.3. Cutaneous Capillary Blood Flow (Figure 3)

In the experimental group, baseline measurement showed a cutaneous capillary blood flow at 28.9 ± 16.3 AU with a similar measurement of 29.4 ± 12.8 AU in the placebo group (*P* > 0.05). From minute 1 to minute 10 after ESWT application, blood flow showed an insignificant increase. However, after the second application of ESWT cutaneous capillary blood flow increased significantly (126.6 ± 60.5 AU; *P* > 0.05 versus baseline) and remained significantly elevated for 20 minutes with a second peak after the third application of ESWT (121.8 ± 54.5 AU; *P* > 0.05 versus baseline). In contrast, cutaneous capillary blood velocity in the placebo group remained unchanged.

## 4. Discussion

Fractionated repetitive ESWT (frESWT) applied to a skin area on the hind limb of the rat results in a prolonged skin microcirculation enhancement. Blood flow and tissue oxygen saturation significantly increased after the first application of high-energy ESWT. Further, the effect was multiplied after the second and the third application. Tissue oxygen saturation showed a significant increase even 80 minutes after the first treatment.

In recent studies, it was demonstrated that a single application of ESWT improves microcirculatory parameters of the skin and was therefore thought to be of special interest in various skin diseases and wounds. High-energy focused ESWT, meaning more than 0.15 J/mm^2^, showed superior results compared with low-energy ESWT. Unfortunately, all of these effects had a limited duration (Tobias Kisch, submitted manuscript, February 2015; Robert Kraemer, submitted manuscript, May 2015). Nevertheless, clinical studies revealed positive effects after a single application of ESWT.

In a prospective randomized trial including 44 patients, ESWT was used with 0.1 mJ/mm^2^ and 100 impulses/cm^2^ on superficial second-degree burn wounds. Epithelialization was enhanced by a mean of 2 days [11]. Same procedure was performed in standardized skin graft donor site model. Reepithelialization was found to be accelerated after a single application of ESWT [12].

On the other hand, a single application of ESWT was compared with the delivery of two and three applications of ESWT in a full-thickness burn wound in the ear of hairless mice. A significantly higher reduction of nonperfused areas was found in the group that received repetitive ESWT [24]. Moreover, Cantaldo et al. found a significant increase in wound healing and vascular density after the second treatment of wounds in mice, but not after the first treatment [25]. However, treatment intervals in both studies were two or more days which may have limited the size of the positive effects.

Wound healing depends on the existence of a vascular network and the nutrition of ischemic tissues, but other factors play a critical role in tissue healing, too. The migration and proliferation of pluripotent cells due to the presence of cytokines are regularly emphasized. Therefore, new therapeutic strategies with growth factor application or platelet-rich plasma (PRP) or even cell based strategies aim to improve wound healing [26]. Recently, Qui et al. could show that ESWT had a positive impact on the mobilization of mesenchymal stem cells (MSC) in a diabetic rat model [27]. Furthermore, effects of ESWT on connective tissue have also been shown [5, 28]. Appearance of cellulite was improved after six to eight applications [16, 29], burn scars were softened [15], and systemic sclerosis was significantly reduced. In a prospective study including 30 patients, skin structure of systemic sclerosis was found to be more regular after a single application of ESWT. Moreover, improved skin vascularization was shown after 90 days. Endothelial progenitor cells were demonstrated to be elevated, while von Willebrand factor (vWF), vascular endothelial growth factor (VEGF), intracellular adhesion molecule-1, and monocyte chemotactic protein-1 (MCP-1) did not rise 30 and 60 days after treatment [18].

Other studies revealed an enhanced release of VEGF and NO after ESWT, resulting in an improvement of tissue vascularization [30, 31]. Consequently, fractionated repetitive application may also boost proangiogenic factor release, resulting in a sustained effect. In this context, prolonged shear stress is known to increase angiogenic sprouting [32]. Thus, it is thought that the mechanical stimulus of ESWT in combination with the improved blood flow may have additive effects. ESWT has been shown to impact the mechanosensory complex formation involving VEGFR-2, VE-cadherin, and PECAM-1 [6, 33].

With regard to our study results, fractionated repetitive ESWT may enhance skin vascularization to a greater degree than single application. In further studies, shorter intervals should be compared with longer intervals and a single application of ESWT. Application of fractionated repetitive ESWT on perfusion and healing of other tissues, for example, muscle and bone, should also be investigated in further clinical and experimental studies.

## 5. Conclusion

ESWT can inhibit skin inflammation, improve skin smoothness, and enhance wound healing. High-energy ESWT improves blood flow and tissue oxygen saturation. Since fractionated repetitive ESWT with short intervals prolongs these effects, it may be of special interest for the treatment of various types of skin diseases and skin damages. Fractionated repetitive ESWT increases the amount of energy delivered to the tissue; therefore, it has the potential to be of high clinical and experimental relevance.

## Figures and Tables

**Figure 1:**
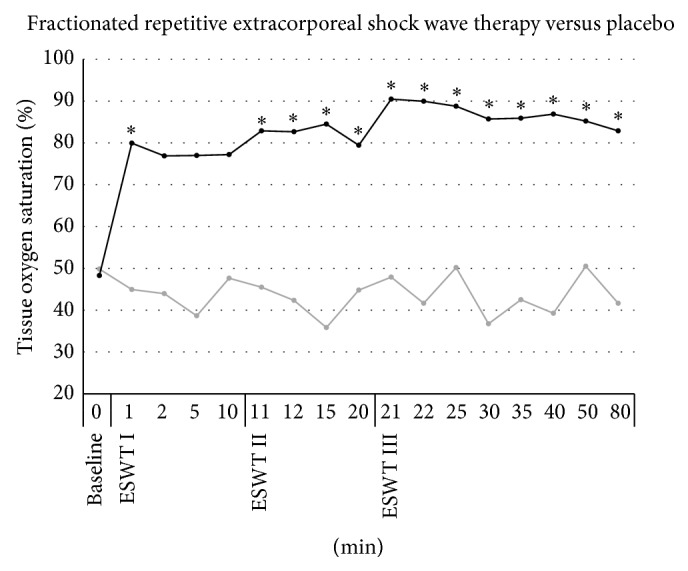
Microcirculatory effects of frESWT on cutaneous tissue oxygen saturation. One minute after the first application of ESWT (black) tissue oxygen saturation significantly increased by 65.6% and remained elevated until the second ESWT application. Tissue oxygen saturation showed a boost to the highest extent after the third application of ESWT and remained significantly increased until the end of measuring at 80 minutes. Placebo (grey) showed no significant changes in tissue oxygen saturation.

**Figure 2:**
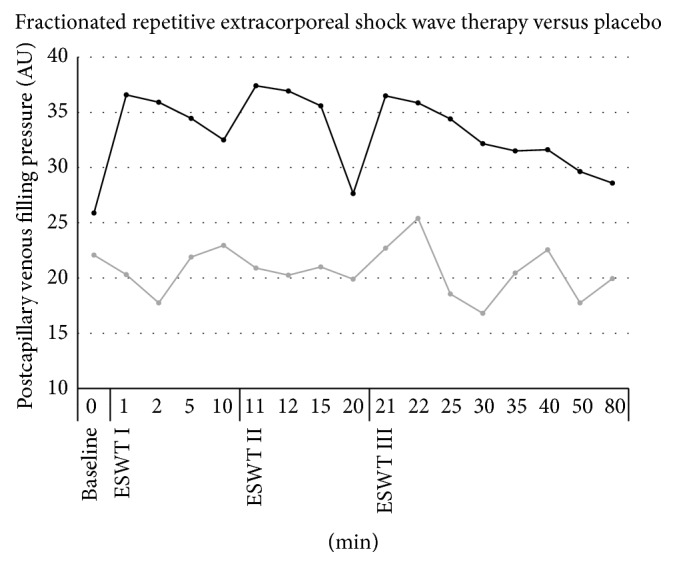
Microcirculatory effects of frESWT on cutaneous postcapillary venous filling pressure. One minute after each application of frESWT (black) postcapillary venous filling pressure increased, statistically insignificant, respectively, and decreased to baseline 20 minutes after application. Placebo (grey) showed no relevant changes from baseline.

**Figure 3:**
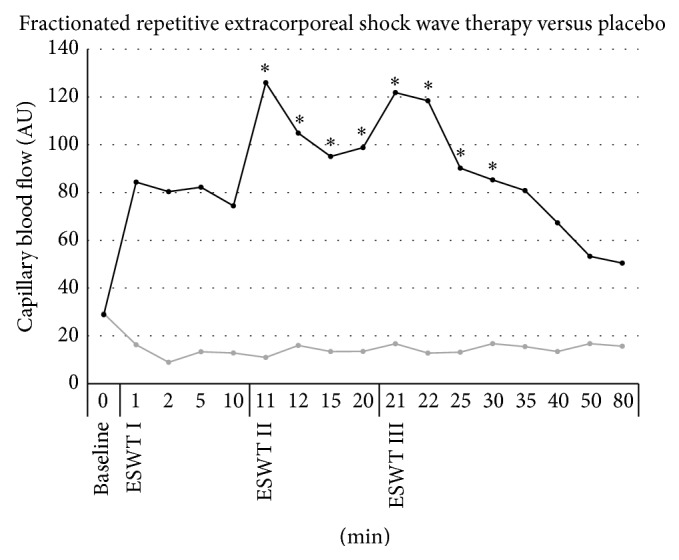
Microcirculatory effects of frESWT on cutaneous capillary blood flow. Blood flow was insignificantly increased from minute 1 to minute 10 after ESWT (black) application. After the second application of ESWT cutaneous capillary blood flow increased significantly and remained significantly elevated for 20 minutes with a second peak after the third application. Placebo (grey) did not show significant changes in blood flow.
